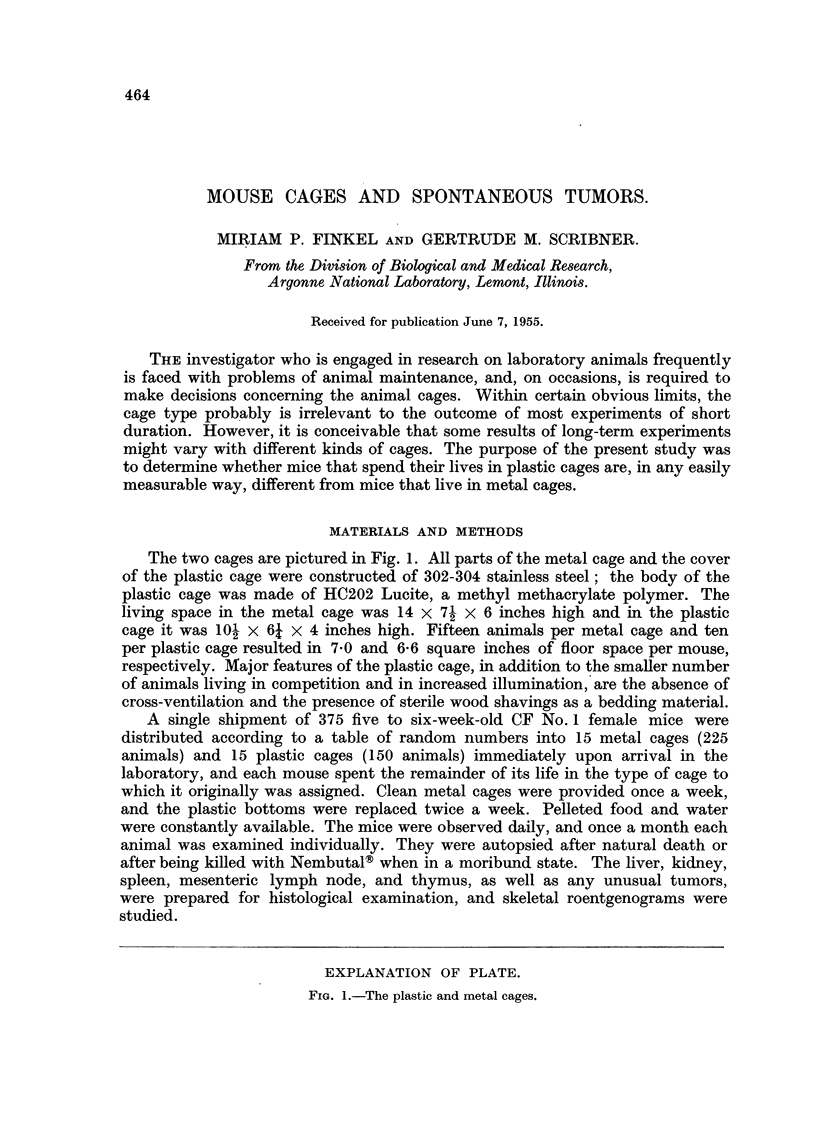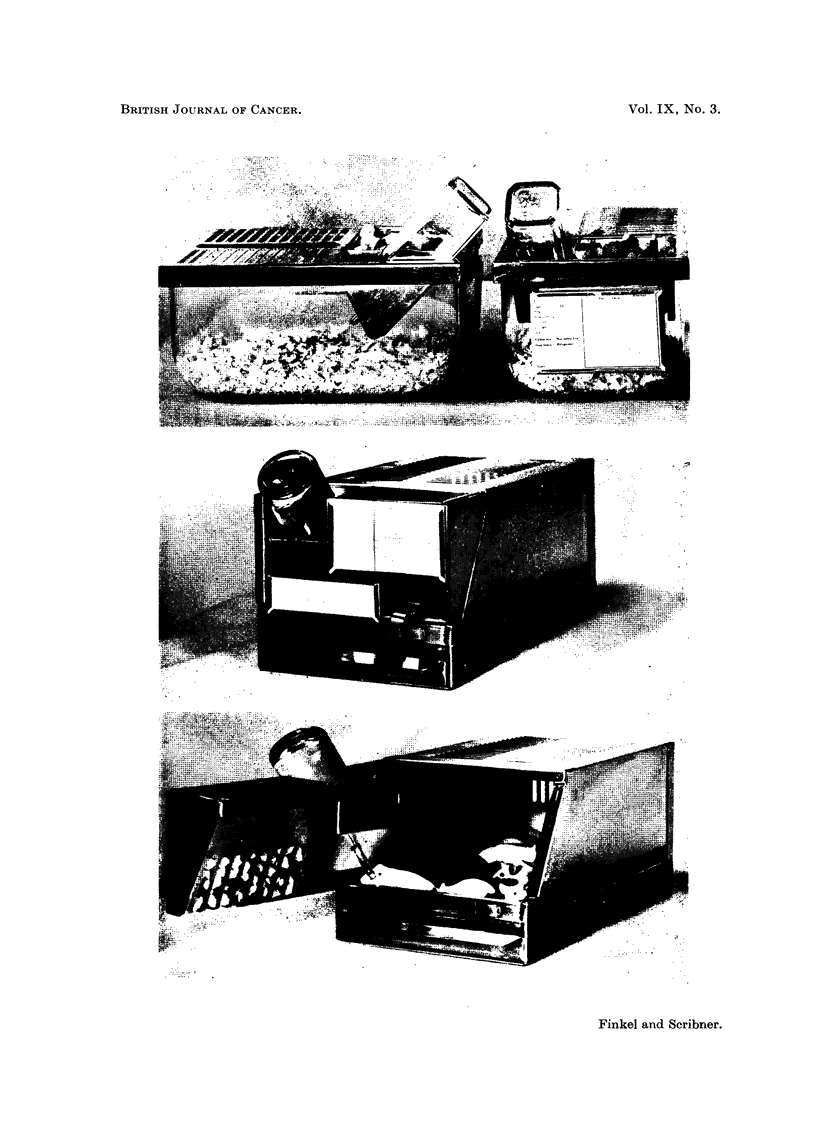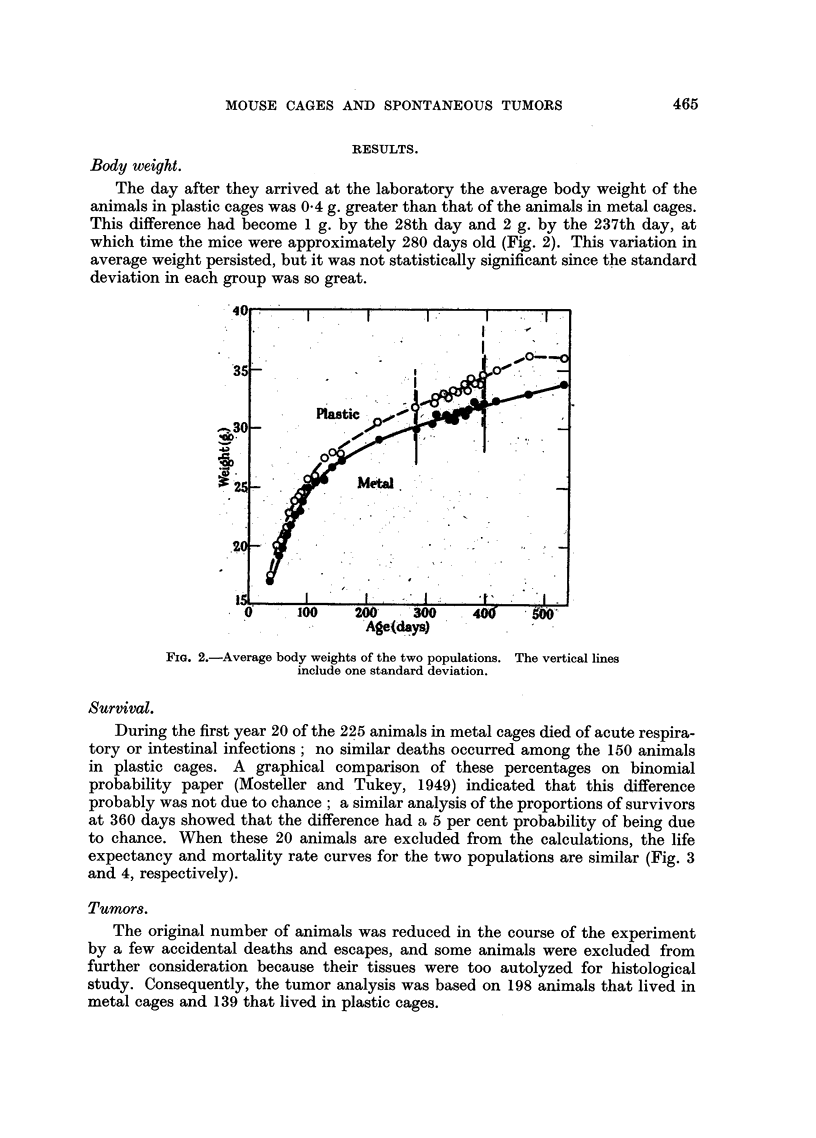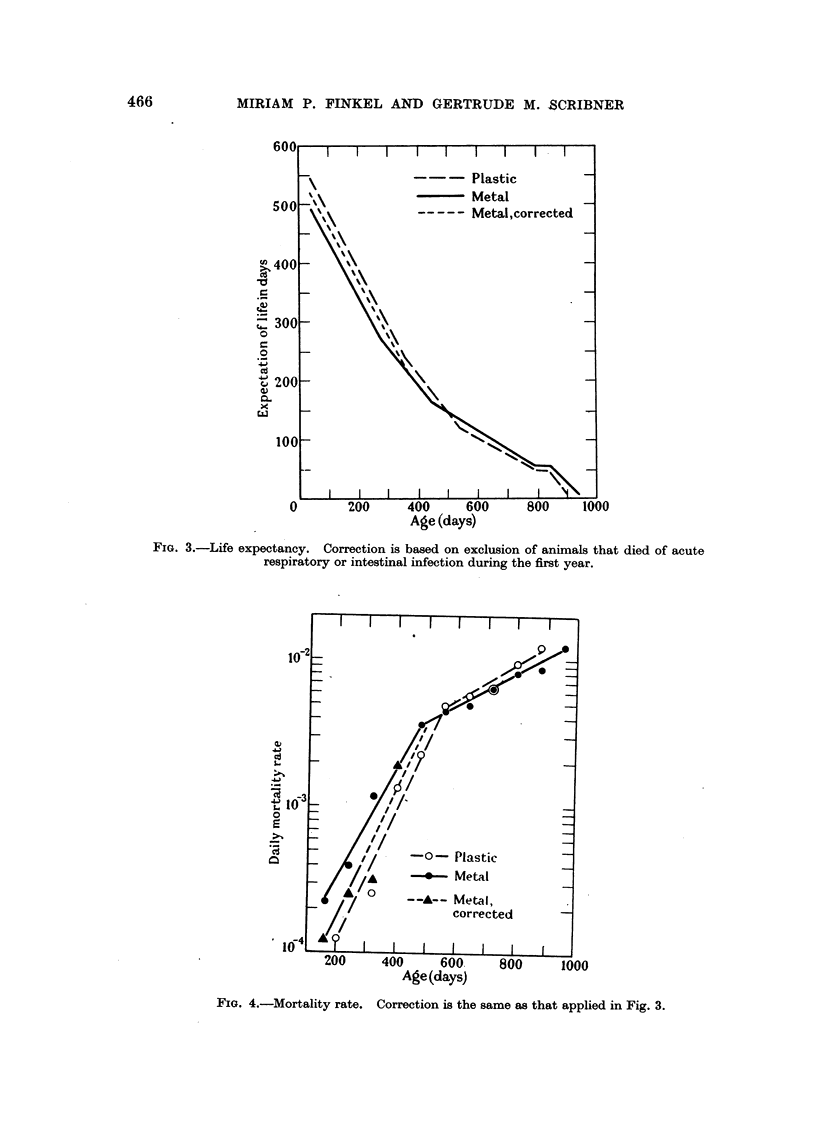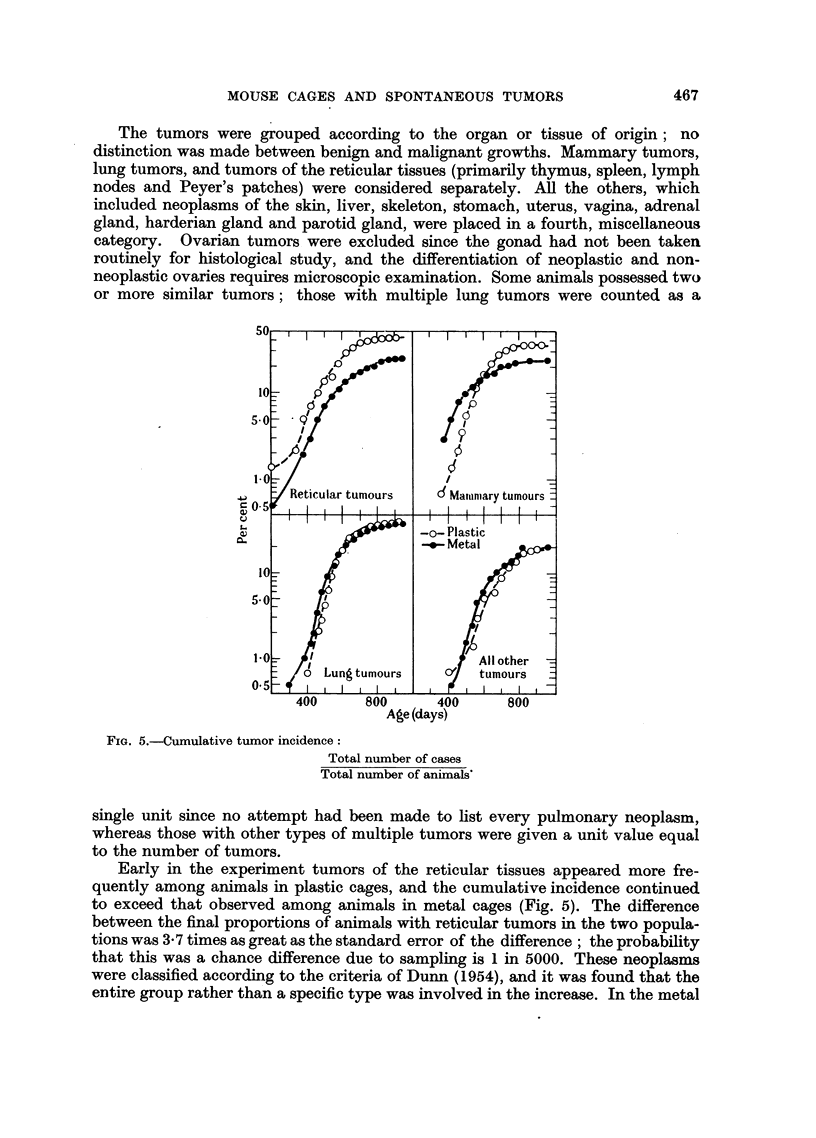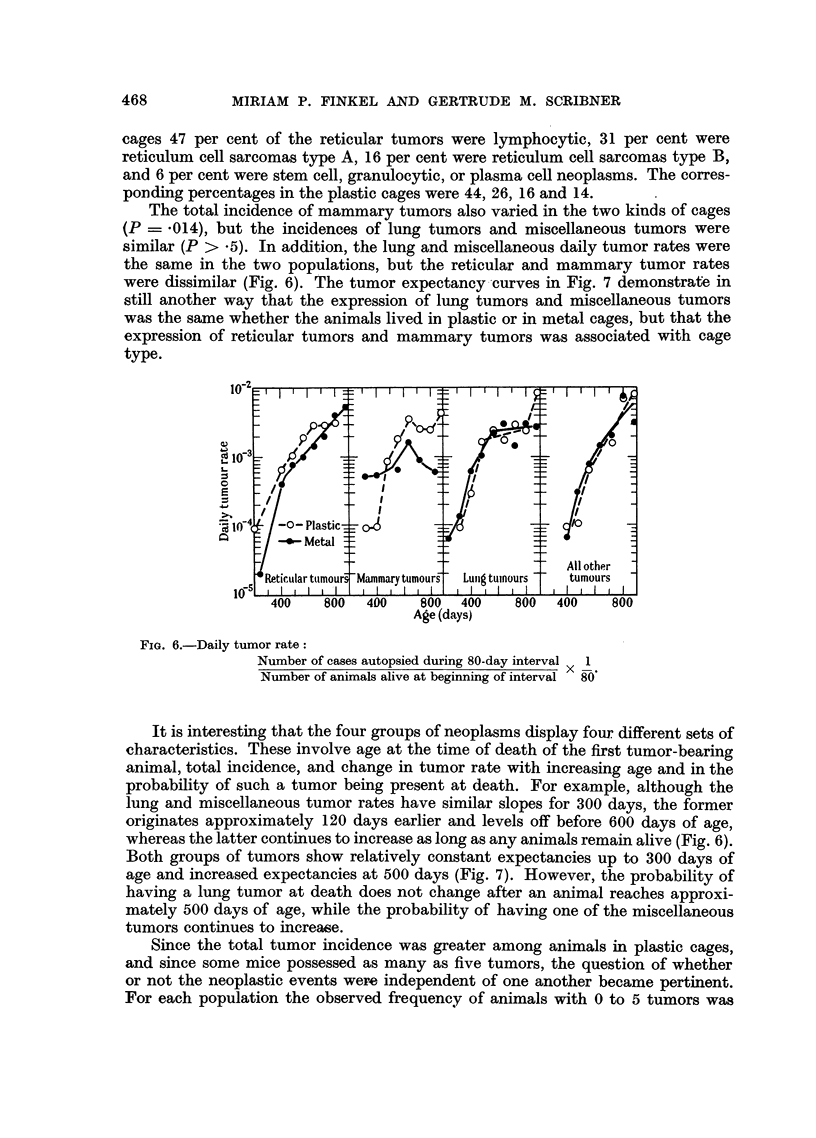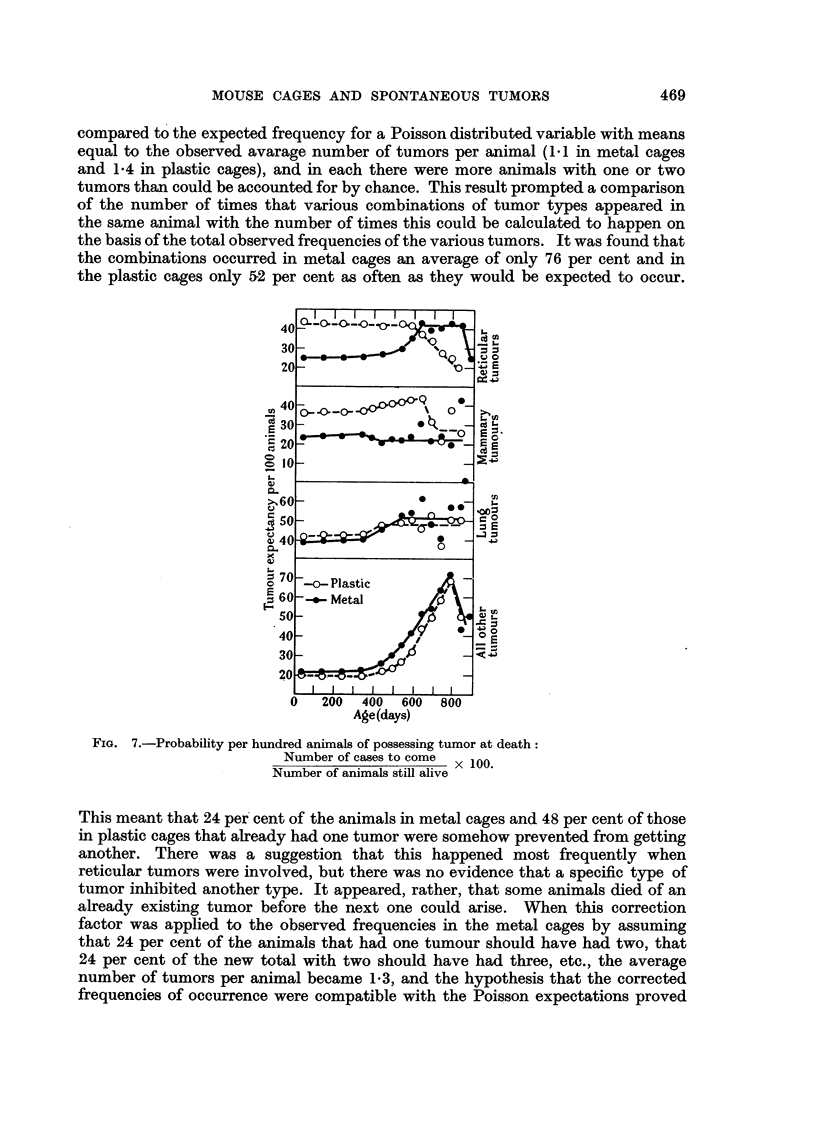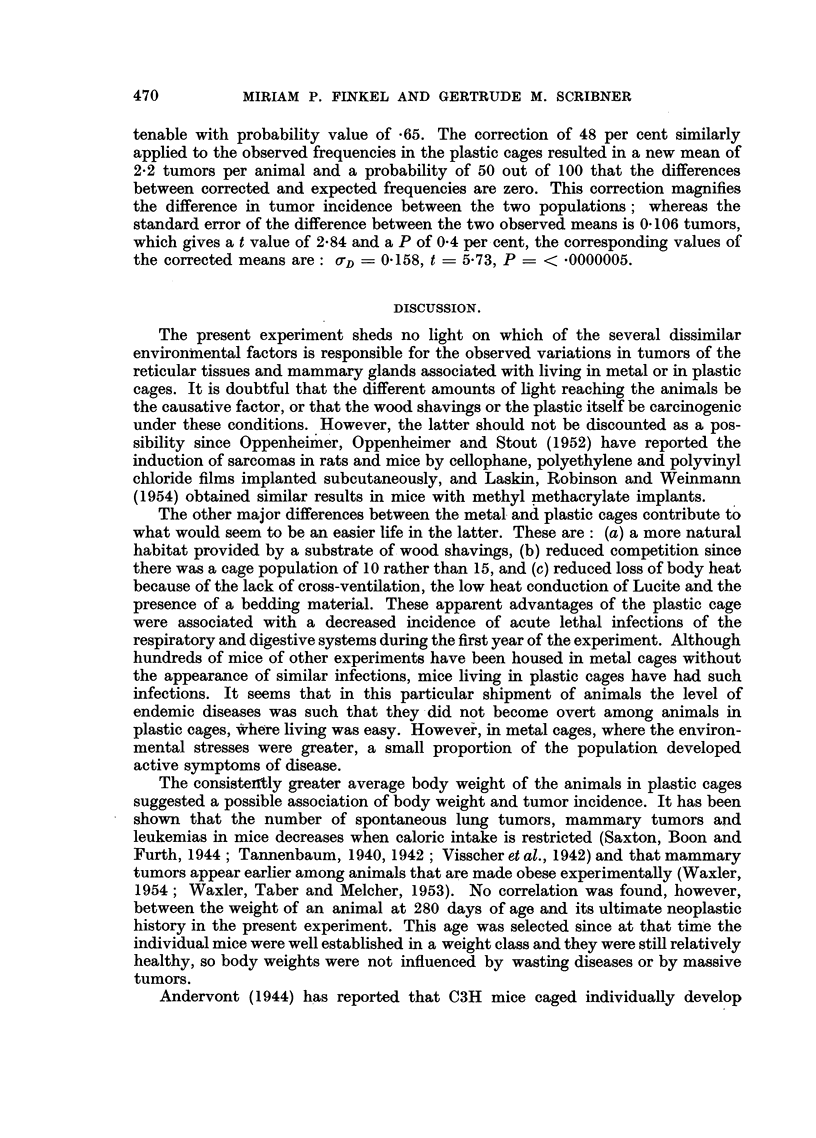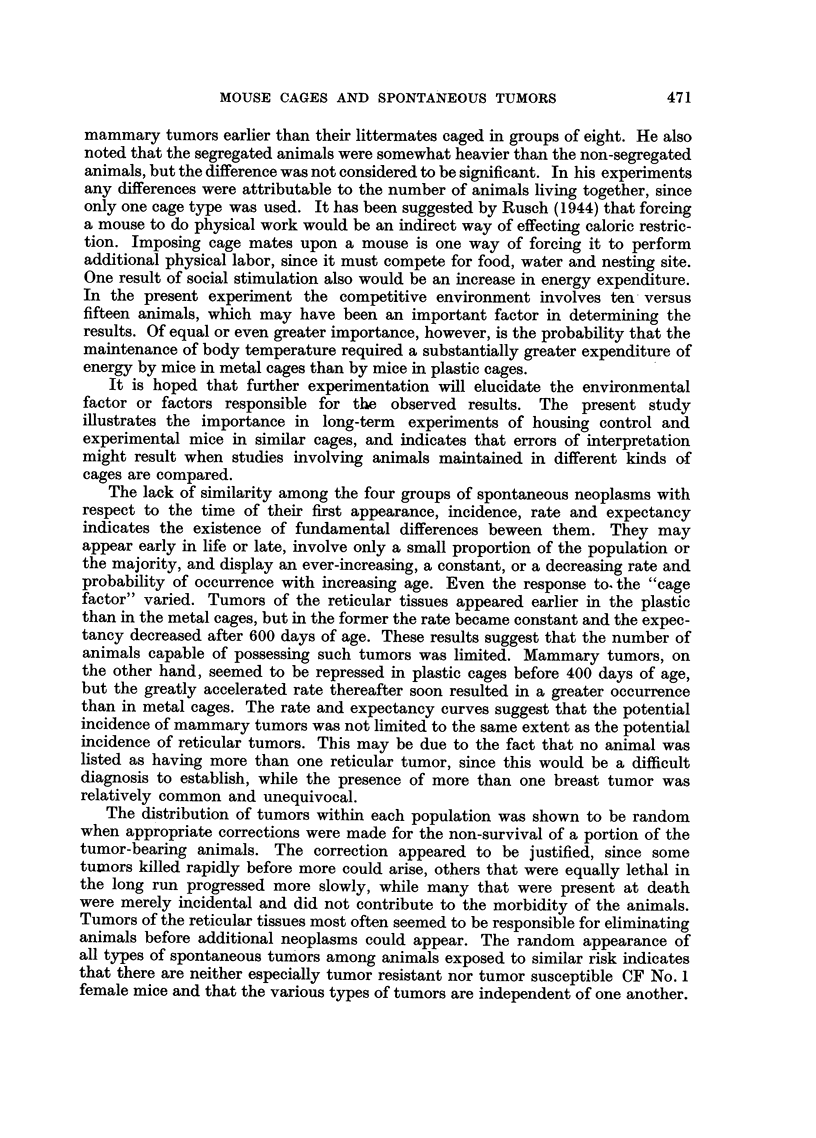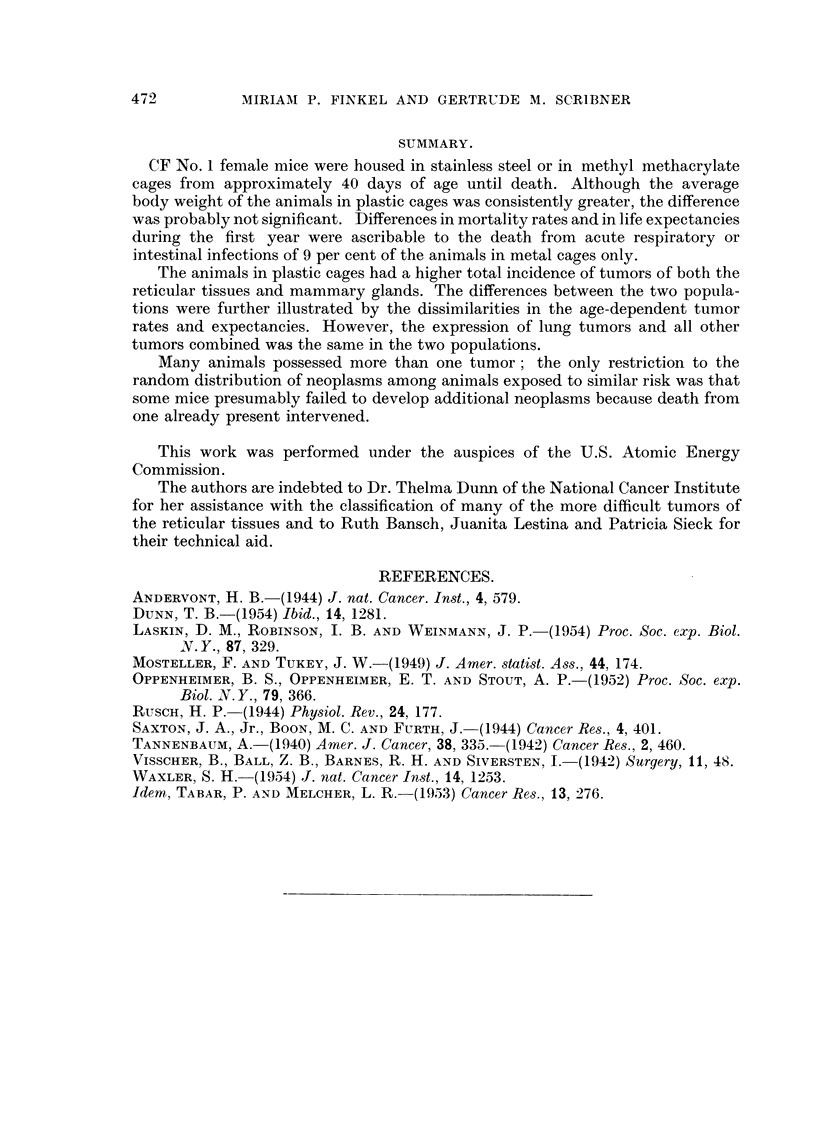# Mouse Cages and Spontaneous Tumors

**DOI:** 10.1038/bjc.1955.48

**Published:** 1955-09

**Authors:** Miriam P. Finkel, Gertrude M. Scribner

## Abstract

**Images:**


### MOUSE CAGES AND SPONTANEOUS TUMORS.

MIRIAM P. FINKEL AND GERTRUDE M. SCRIBNER.

*From the Division of Biological and Medical Research, Argonne National Laboratory, Lemont, Illinois.*

Received for publication June 7, 1955.

THE investigator who is engaged in research on laboratory animals frequently is faced with problems of animal maintenance, and, on occasions, is required to make decisions concerning the animal cages. Within certain obvious limits, the cage type probably is irrelevant to the outcome of most experiments of short duration. However, it is conceivable that some results of long-term experiments might vary with different kinds of cages. The purpose of the present study was to determine whether mice that spend their lives in plastic cages are, in any easily measurable way, different from mice that live in metal cages.

### MATERIALS AND METHODS

The two cages are pictured in Fig. 1. All parts of the metal cage and the cover of the plastic cage were constructed of 302-304 stainless steel; the body of the plastic cage was made of HC202 Lucite, a methyl methacrylate polymer. The living space in the metal cage was $14 \times 7\frac{1}{2} \times 6$ inches high and in the plastic cage it was $10\frac{1}{2} \times 6\frac{1}{4} \times 4$ inches high. Fifteen animals per metal cage and ten per plastic cage resulted in 7·0 and 6·6 square inches of floor space per mouse, respectively. Major features of the plastic cage, in addition to the smaller number of animals living in competition and in increased illumination, are the absence of cross-ventilation and the presence of sterile wood shavings as a bedding material.

A single shipment of 375 five to six-week-old CF No. 1 female mice were distributed according to a table of random numbers into 15 metal cages (225 animals) and 15 plastic cages (150 animals) immediately upon arrival in the laboratory, and each mouse spent the remainder of its life in the type of cage to which it originally was assigned. Clean metal cages were provided once a week, and the plastic bottoms were replaced twice a week. Pelleted food and water were constantly available. The mice were observed daily, and once a month each animal was examined individually. They were autopsied after natural death or after being killed with Nembutal® when in a moribund state. The liver, kidney, spleen, mesenteric lymph node, and thymus, as well as any unusual tumors, were prepared for histological examination, and skeletal roentgenograms were studied.

---

EXPLANATION OF PLATE.

FIG. 1.—The plastic and metal cages.

Finkel and Scribner.

### RESULTS.

*Body weight.*

The day after they arrived at the laboratory the average body weight of the animals in plastic cages was 0·4 g. greater than that of the animals in metal cages. This difference had become 1 g. by the 28th day and 2 g. by the 237th day, at which time the mice were approximately 280 days old (Fig. 2). This variation in average weight persisted, but it was not statistically significant since the standard deviation in each group was so great.

FIG. 2.—Average body weights of the two populations. The vertical lines include one standard deviation.

*Survival.*

During the first year 20 of the 225 animals in metal cages died of acute respiratory or intestinal infections ; no similar deaths occurred among the 150 animals in plastic cages. A graphical comparison of these percentages on binomial probability paper (Mosteller and Tukey, 1949) indicated that this difference probably was not due to chance ; a similar analysis of the proportions of survivors at 360 days showed that the difference had a 5 per cent probability of being due to chance. When these 20 animals are excluded from the calculations, the life expectancy and mortality rate curves for the two populations are similar (Fig. 3 and 4, respectively).

*Tumors.*

The original number of animals was reduced in the course of the experiment by a few accidental deaths and escapes, and some animals were excluded from further consideration because their tissues were too autolyzed for histological study. Consequently, the tumor analysis was based on 198 animals that lived in metal cages and 139 that lived in plastic cages.

FIG. 3.—Life expectancy. Correction is based on exclusion of animals that died of acute respiratory or intestinal infection during the first year.

FIG. 4.—Mortality rate. Correction is the same as that applied in Fig. 3.

The tumors were grouped according to the organ or tissue of origin ; no distinction was made between benign and malignant growths. Mammary tumors, lung tumors, and tumors of the reticular tissues (primarily thymus, spleen, lymph nodes and Peyer's patches) were considered separately. All the others, which included neoplasms of the skin, liver, skeleton, stomach, uterus, vagina, adrenal gland, harderian gland and parotid gland, were placed in a fourth, miscellaneous category. Ovarian tumors were excluded since the gonad had not been taken routinely for histological study, and the differentiation of neoplastic and non-neoplastic ovaries requires microscopic examination. Some animals possessed two or more similar tumors ; those with multiple lung tumors were counted as a

FIG. 5.—Cumulative tumor incidence :

$$\frac{\text{Total number of cases}}{\text{Total number of animals}}.$$

single unit since no attempt had been made to list every pulmonary neoplasm, whereas those with other types of multiple tumors were given a unit value equal to the number of tumors.

Early in the experiment tumors of the reticular tissues appeared more frequently among animals in plastic cages, and the cumulative incidence continued to exceed that observed among animals in metal cages (Fig. 5). The difference between the final proportions of animals with reticular tumors in the two populations was 3·7 times as great as the standard error of the difference ; the probability that this was a chance difference due to sampling is 1 in 5000. These neoplasms were classified according to the criteria of Dunn (1954), and it was found that the entire group rather than a specific type was involved in the increase. In the metal cages 47 per cent of the reticular tumors were lymphocytic, 31 per cent were reticulum cell sarcomas type A, 16 per cent were reticulum cell sarcomas type B, and 6 per cent were stem cell, granulocytic, or plasma cell neoplasms. The corresponding percentages in the plastic cages were 44, 26, 16 and 14.

The total incidence of mammary tumors also varied in the two kinds of cages ($P = \cdot 014$), but the incidences of lung tumors and miscellaneous tumors were similar ($P > \cdot 5$). In addition, the lung and miscellaneous daily tumor rates were the same in the two populations, but the reticular and mammary tumor rates were dissimilar (Fig. 6). The tumor expectancy curves in Fig. 7 demonstrate in still another way that the expression of lung tumors and miscellaneous tumors was the same whether the animals lived in plastic or in metal cages, but that the expression of reticular tumors and mammary tumors was associated with cage type.

FIG. 6.—Daily tumor rate :

$$\frac{\text{Number of cases autopsied during 80-day interval}}{\text{Number of animals alive at beginning of interval}} \times \frac{1}{80}.$$

It is interesting that the four groups of neoplasms display four different sets of characteristics. These involve age at the time of death of the first tumor-bearing animal, total incidence, and change in tumor rate with increasing age and in the probability of such a tumor being present at death. For example, although the lung and miscellaneous tumor rates have similar slopes for 300 days, the former originates approximately 120 days earlier and levels off before 600 days of age, whereas the latter continues to increase as long as any animals remain alive (Fig. 6). Both groups of tumors show relatively constant expectancies up to 300 days of age and increased expectancies at 500 days (Fig. 7). However, the probability of having a lung tumor at death does not change after an animal reaches approximately 500 days of age, while the probability of having one of the miscellaneous tumors continues to increase.

Since the total tumor incidence was greater among animals in plastic cages, and since some mice possessed as many as five tumors, the question of whether or not the neoplastic events were independent of one another became pertinent. For each population the observed frequency of animals with 0 to 5 tumors was compared to the expected frequency for a Poisson distributed variable with means equal to the observed avarage number of tumors per animal (1·1 in metal cages and 1·4 in plastic cages), and in each there were more animals with one or two tumors than could be accounted for by chance. This result prompted a comparison of the number of times that various combinations of tumor types appeared in the same animal with the number of times this could be calculated to happen on the basis of the total observed frequencies of the various tumors. It was found that the combinations occurred in metal cages an average of only 76 per cent and in the plastic cages only 52 per cent as often as they would be expected to occur.

FIG. 7.—Probability per hundred animals of possessing tumor at death :

$$\frac{\text{Number of cases to come}}{\text{Number of animals still alive}} \times 100.$$

This meant that 24 per cent of the animals in metal cages and 48 per cent of those in plastic cages that already had one tumor were somehow prevented from getting another. There was a suggestion that this happened most frequently when reticular tumors were involved, but there was no evidence that a specific type of tumor inhibited another type. It appeared, rather, that some animals died of an already existing tumor before the next one could arise. When this correction factor was applied to the observed frequencies in the metal cages by assuming that 24 per cent of the animals that had one tumour should have had two, that 24 per cent of the new total with two should have had three, etc., the average number of tumors per animal became 1·3, and the hypothesis that the corrected frequencies of occurrence were compatible with the Poisson expectations proved tenable with probability value of ·65. The correction of 48 per cent similarly applied to the observed frequencies in the plastic cages resulted in a new mean of 2·2 tumors per animal and a probability of 50 out of 100 that the differences between corrected and expected frequencies are zero. This correction magnifies the difference in tumor incidence between the two populations; whereas the standard error of the difference between the two observed means is 0·106 tumors, which gives a $t$ value of 2·84 and a $P$ of 0·4 per cent, the corresponding values of the corrected means are: $\sigma_D = 0{\cdot}158$, $t = 5{\cdot}73$, $P = < {\cdot}0000005$.

### DISCUSSION.

The present experiment sheds no light on which of the several dissimilar environmental factors is responsible for the observed variations in tumors of the reticular tissues and mammary glands associated with living in metal or in plastic cages. It is doubtful that the different amounts of light reaching the animals be the causative factor, or that the wood shavings or the plastic itself be carcinogenic under these conditions. However, the latter should not be discounted as a possibility since Oppenheimer, Oppenheimer and Stout (1952) have reported the induction of sarcomas in rats and mice by cellophane, polyethylene and polyvinyl chloride films implanted subcutaneously, and Laskin, Robinson and Weinmann (1954) obtained similar results in mice with methyl methacrylate implants.

The other major differences between the metal and plastic cages contribute to what would seem to be an easier life in the latter. These are: (*a*) a more natural habitat provided by a substrate of wood shavings, (b) reduced competition since there was a cage population of 10 rather than 15, and (*c*) reduced loss of body heat because of the lack of cross-ventilation, the low heat conduction of Lucite and the presence of a bedding material. These apparent advantages of the plastic cage were associated with a decreased incidence of acute lethal infections of the respiratory and digestive systems during the first year of the experiment. Although hundreds of mice of other experiments have been housed in metal cages without the appearance of similar infections, mice living in plastic cages have had such infections. It seems that in this particular shipment of animals the level of endemic diseases was such that they did not become overt among animals in plastic cages, where living was easy. However, in metal cages, where the environmental stresses were greater, a small proportion of the population developed active symptoms of disease.

The consistently greater average body weight of the animals in plastic cages suggested a possible association of body weight and tumor incidence. It has been shown that the number of spontaneous lung tumors, mammary tumors and leukemias in mice decreases when caloric intake is restricted (Saxton, Boon and Furth, 1944; Tannenbaum, 1940, 1942; Visscher *et al.*, 1942) and that mammary tumors appear earlier among animals that are made obese experimentally (Waxler, 1954; Waxler, Taber and Melcher, 1953). No correlation was found, however, between the weight of an animal at 280 days of age and its ultimate neoplastic history in the present experiment. This age was selected since at that time the individual mice were well established in a weight class and they were still relatively healthy, so body weights were not influenced by wasting diseases or by massive tumors.

Andervont (1944) has reported that C3H mice caged individually develop mammary tumors earlier than their littermates caged in groups of eight. He also noted that the segregated animals were somewhat heavier than the non-segregated animals, but the difference was not considered to be significant. In his experiments any differences were attributable to the number of animals living together, since only one cage type was used. It has been suggested by Rusch (1944) that forcing a mouse to do physical work would be an indirect way of effecting caloric restriction. Imposing cage mates upon a mouse is one way of forcing it to perform additional physical labor, since it must compete for food, water and nesting site. One result of social stimulation also would be an increase in energy expenditure. In the present experiment the competitive environment involves ten versus fifteen animals, which may have been an important factor in determining the results. Of equal or even greater importance, however, is the probability that the maintenance of body temperature required a substantially greater expenditure of energy by mice in metal cages than by mice in plastic cages.

It is hoped that further experimentation will elucidate the environmental factor or factors responsible for the observed results. The present study illustrates the importance in long-term experiments of housing control and experimental mice in similar cages, and indicates that errors of interpretation might result when studies involving animals maintained in different kinds of cages are compared.

The lack of similarity among the four groups of spontaneous neoplasms with respect to the time of their first appearance, incidence, rate and expectancy indicates the existence of fundamental differences beween them. They may appear early in life or late, involve only a small proportion of the population or the majority, and display an ever-increasing, a constant, or a decreasing rate and probability of occurrence with increasing age. Even the response to the "cage factor" varied. Tumors of the reticular tissues appeared earlier in the plastic than in the metal cages, but in the former the rate became constant and the expectancy decreased after 600 days of age. These results suggest that the number of animals capable of possessing such tumors was limited. Mammary tumors, on the other hand, seemed to be repressed in plastic cages before 400 days of age, but the greatly accelerated rate thereafter soon resulted in a greater occurrence than in metal cages. The rate and expectancy curves suggest that the potential incidence of mammary tumors was not limited to the same extent as the potential incidence of reticular tumors. This may be due to the fact that no animal was listed as having more than one reticular tumor, since this would be a difficult diagnosis to establish, while the presence of more than one breast tumor was relatively common and unequivocal.

The distribution of tumors within each population was shown to be random when appropriate corrections were made for the non-survival of a portion of the tumor-bearing animals. The correction appeared to be justified, since some tumors killed rapidly before more could arise, others that were equally lethal in the long run progressed more slowly, while many that were present at death were merely incidental and did not contribute to the morbidity of the animals. Tumors of the reticular tissues most often seemed to be responsible for eliminating animals before additional neoplasms could appear. The random appearance of all types of spontaneous tumors among animals exposed to similar risk indicates that there are neither especially tumor resistant nor tumor susceptible CF No. 1 female mice and that the various types of tumors are independent of one another.

SUMMARY.

CF No. 1 female mice were housed in stainless steel or in methyl methacrylate cages from approximately 40 days of age until death. Although the average body weight of the animals in plastic cages was consistently greater, the difference was probably not significant. Differences in mortality rates and in life expectancies during the first year were ascribable to the death from acute respiratory or intestinal infections of 9 per cent of the animals in metal cages only.

The animals in plastic cages had a higher total incidence of tumors of both the reticular tissues and mammary glands. The differences between the two populations were further illustrated by the dissimilarities in the age-dependent tumor rates and expectancies. However, the expression of lung tumors and all other tumors combined was the same in the two populations.

Many animals possessed more than one tumor ; the only restriction to the random distribution of neoplasms among animals exposed to similar risk was that some mice presumably failed to develop additional neoplasms because death from one already present intervened.

This work was performed under the auspices of the U.S. Atomic Energy Commission.

The authors are indebted to Dr. Thelma Dunn of the National Cancer Institute for her assistance with the classification of many of the more difficult tumors of the reticular tissues and to Ruth Bansch, Juanita Lestina and Patricia Sieck for their technical aid.

REFERENCES.

Andervont, H. B.—(1944) *J. nat. Cancer. Inst.*, **4**, 579.
Dunn, T. B.—(1954) *Ibid.*, **14**, 1281.
Laskin, D. M., Robinson, I. B. and Weinmann, J. P.—(1954) *Proc. Soc. exp. Biol. N.Y.*, **87**, 329.
Mosteller, F. and Tukey, J. W.—(1949) *J. Amer. statist. Ass.*, **44**, 174.
Oppenheimer, B. S., Oppenheimer, E. T. and Stout, A. P.—(1952) *Proc. Soc. exp. Biol. N.Y.*, **79**, 366.
Rusch, H. P.—(1944) *Physiol. Rev.*, **24**, 177.
Saxton, J. A., Jr., Boon, M. C. and Furth, J.—(1944) *Cancer Res.*, **4**, 401.
Tannenbaum, A.—(1940) *Amer. J. Cancer*, **38**, 335.—(1942) *Cancer Res.*, **2**, 460.
Visscher, B., Ball, Z. B., Barnes, R. H. and Siversten, I.—(1942) *Surgery*, **11**, 48.
Waxler, S. H.—(1954) *J. nat. Cancer Inst.*, **14**, 1253.
*Idem*, Tabar, P. and Melcher, L. R.—(1953) *Cancer Res.*, **13**, 276.